# Genome-wide mapping of i-motifs reveals their association with transcription regulation in live human cells

**DOI:** 10.1093/nar/gkad626

**Published:** 2023-08-02

**Authors:** Irene Zanin, Emanuela Ruggiero, Giulia Nicoletto, Sara Lago, Ilaria Maurizio, Irene Gallina, Sara N Richter

**Affiliations:** Department of Molecular Medicine, University of Padua, 35121 Padua, Italy; Department of Molecular Medicine, University of Padua, 35121 Padua, Italy; Department of Molecular Medicine, University of Padua, 35121 Padua, Italy; Department of Cellular, Computational and Integrative Biology (CIBIO), University of Trento, 38123 Trento, Italy; Department of Molecular Medicine, University of Padua, 35121 Padua, Italy; Department of Molecular Medicine, University of Padua, 35121 Padua, Italy; Department of Molecular Medicine, University of Padua, 35121 Padua, Italy; Microbiology and Virology Unit, Padua University Hospital, 35121 Padua, Italy

## Abstract

i-Motifs (iMs) are four-stranded DNA structures that form at cytosine (C)-rich sequences in acidic conditions *in vitro*. Their formation in cells is still under debate. We performed CUT&Tag sequencing using the anti-iM antibody iMab and showed that iMs form within the human genome in live cells. We mapped iMs in two human cell lines and recovered C-rich sequences that were confirmed to fold into iMs *in vitro*. We found that iMs in cells are mainly present at actively transcribing gene promoters, in open chromatin regions, they overlap with R-loops, and their abundance and distribution are specific to each cell type. iMs with both long and short C-tracts were recovered, further extending the relevance of iMs. By simultaneously mapping G-quadruplexes (G4s), which form at guanine-rich regions, and comparing the results with iMs, we proved that the two structures can form in independent regions; however, when both iMs and G4s are present in the same genomic tract, their formation is enhanced. iMs and G4s were mainly found at genes with low and high transcription rates, respectively. Our findings support the *in vivo* formation of iM structures and provide new insights into their interplay with G4s as new regulatory elements in the human genome.

## Graphical Abstract

**Graphical Abstract:**
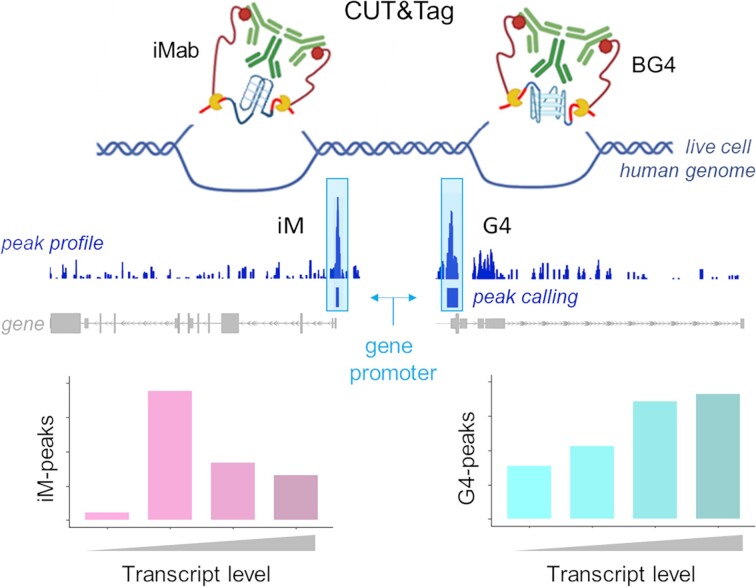

## INTRODUCTION

**Figure 1.**
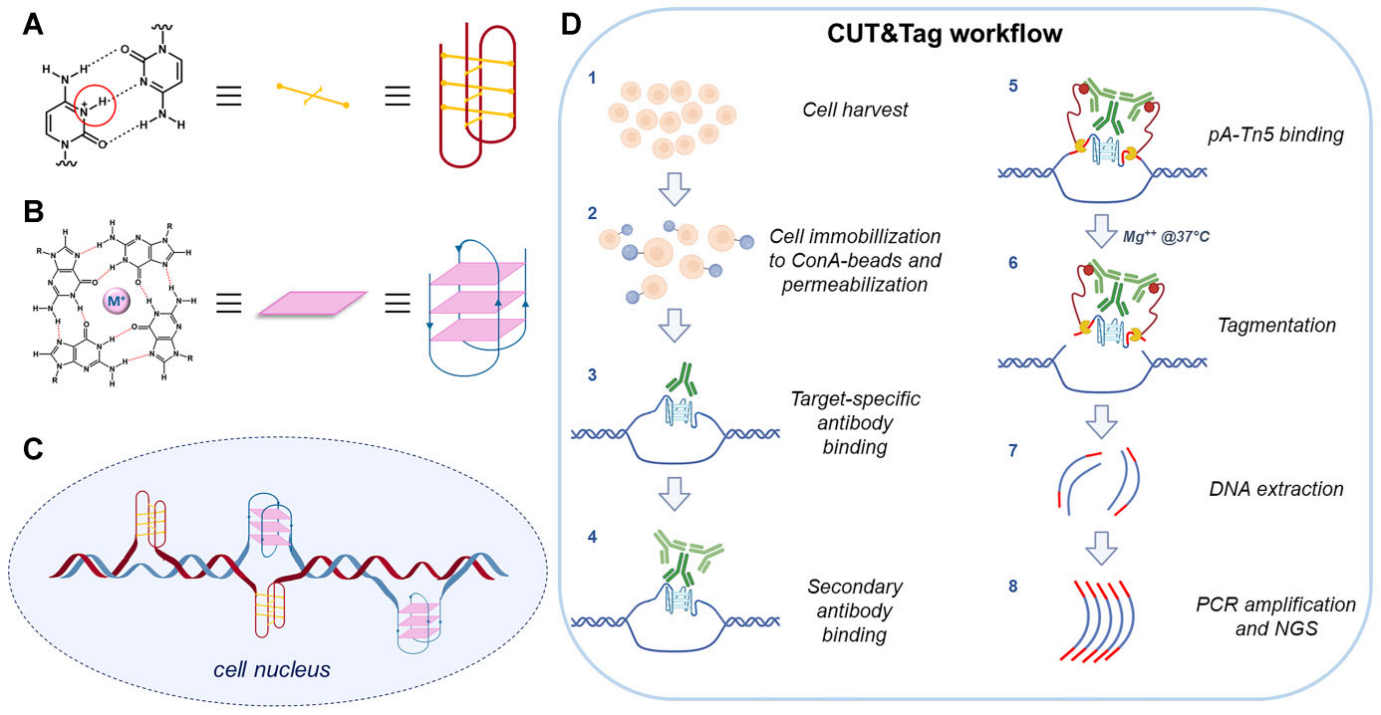
Quadruplex structures and CUT&Tag workflow. (**A**) Two or more intercalated C–C^+^ base pairs form the iM. (**B**) G-quartets, characterized by Hoogsteen H-bonds among four Gs and centrally stabilized by monovalent cations, self-stack to form a G4. (**C**) Cartoon representation of iMs and G4s within the double stranded DNA in the cell nucleus. (**D**) Schematic representation of the CUT&Tag assay workflow (image created with BioRender.com).

G-quadruplexes (G4s) and i-motifs (iMs) are non-canonical nucleic acid structures, alternative to the Watson-Crick double-helix conformation. iMs occur at cytosine (C)-rich regions, when C-C^+^ hemiprotonated base pairs intercalate to build a quadruplex structure (Figure 1A) (1). G4s form at guanine (G)-rich strands when four Gs, linked by Hoogsteen H-bonds, organize into stacked G-quartets stabilized by monovalent cations (Figure 1B) (2). Due to their nature, G4s and iMs can in principle form in the complementary strands of the same genomic region (Figure 1C).

Initial biophysical and molecular biology approaches have characterized G4s as key players in pivotal biological processes, such as transcription, replication, epigenetic modifications, genome instability and recombination (3,4). The development of specific G4-recognizing probes (5–8) has allowed, in the more recent years, G4 detection in cells by imaging and genome-wide sequencing methods. Techniques such as G4-ChIP-seq (9,10) and G4-Cleavage Under Targets & Tagmentation (G4-CUT&Tag) (11,12) have shown that DNA G4 folding in cells is highly dynamic and cell specific. G4s have been shown to be located in open chromatin regions and mostly at promoters, where they have been associated with increased transcription, due to transcription factor specific recognition (10,13).

In contrast, the field of iMs is much less developed: initial studies restricted iM formation mainly to acidic conditions (1,14,15). Subsequent bioinformatic and *in vitro* analyses revealed the presence of iM-forming regions at promoters, UTRs and introns (16) and their formation also at neutral pH (17). Transcription factors, intracellular proteins and epigenetic modifiers are thought to regulate iM stability at physiological pH (18–21). Analysis of iM in cells was made possible by the development of an iM-recognizing antibody (iMab) that selectively targets iMs over G4s and duplex DNA (22). Immunofluorescence-based detection demonstrated that iM formation in the nucleus of human cells is cell cycle-dependent, with the highest iM enrichment at the pre-replication stage (G_1_ phase) (22). In rice, iMab-based immunoprecipitation followed by sequencing (iM-IP-seq) identified iM-folded regions at promoters and untranslated regions, and suggested that iMs contribute to transposable element dynamics (23). More recently, the Christ group reported a genome-wide mapping of iMs in the human genome by immunoprecipitation from sheared chromatin, followed by sequencing, where iMs were found to be abundant and widely distributed throughout the genome (24). Both this and the iM-IP-seq method used fragmentation of the extracted genomic DNA, and the iM-IP-seq also relied on acidic buffers to maintain iM folding, factors that may have affected iM formation and may not reflect conditions in cells. Currently, the only methods that do not require a cell fixation step prior to analysis are CUT&RUN (25) and CUT&Tag (26), allowing the detection of nucleic acid folding under more physiological conditions.

To answer the still open question of where iMs form in the human genome, we performed CUT&Tag with the anti-iM iMab antibody in two human cell lines of embryonic and tumor origin. The analysis was performed in parallel with the anti-G4 BG4 antibody to assess differences in iM/G4 formation and localization within the genome. We demonstrated that iMab, used in the CUT&Tag technique, consistently recognizes iM-forming sequences; these are highly enriched at gene promoters, close to the transcription start site (TSS), but are also detected in non-open chromatin regions. Genes with folded iM at their promoters display transcription rates in the lower expression range, in contrast to G4s. The two different cell lines show specific iM enrichment, confirming that iMs, like G4s (10), are cell-type distinctive features. The two structures form independently, as many of the regions identified are folded in either iM or G4. At the same time, however, when iMs and G4s are present in the same genomic region, they show the highest level of enrichment.

## MATERIALS AND METHODS

### Cell cultures and oligonucleotides

93T449 (WDLPS) cell line (ATCC #CRL-3043) was cultured in RPMI 1640 medium (Gibco, LifeTechnologies, #11875093) supplemented with 10% heat-inactivated fetal bovine serum (FBS) (Gibco, LifeTechnologies, #10270106) and 1× Penicillin–Streptomycin (Gibco, LifeTechnologies, #15140122). Human embryonic kidney (HEK293T) cell line (ATCC #CRL-3216) was cultured in DMEM medium (Gibco, LifeTechnologies, #41965062) supplemented with 10% FBS and 1× Penicillin–Streptomycin. Cells were grown under standard conditions (5% CO_2_, 95% humidity, 37°C). Desalted oligonucleotides were purchased from Sigma-Aldrich (Milan, Italy) and all sequences are reported in Supplementary Table S2.

### BG4 production and purification

BG4 antibody preparation was optimized starting from previously published protocols (11,27,28). The pSANG10-3F-BG4 expression plasmid (Addgene, #55756) was transformed in BL21 competent cells (kindly provided by Prof. A. Loregian, University of Padua, Italy). A single colony was grown in 2× TY added with 2% glucose and 50 μg/ml kanamycin overnight at 37°C. The day after, bacteria were refreshed and grown to OD_600_ = 2. Induction was performed with 0.5 mM IPTG at 18°C for 24 h. Bacteria were spun down at 3500 rpm for 15 min at 4°C, then the pellet was resuspended in 3 ml of TES buffer (50 mM Tris–HCl pH 8, 20% sucrose, 1 mM EDTA, protease inhibitors) and the bacteria suspension was incubated 10 min on ice. 4.5 ml of 1:5 TES buffer with 2 mM MgCl_2_ and benzonase were added before incubation for 30 min at 4°C. Bacterial cells were pelleted at 8000 g for 20 min and the supernatant was incubated on HisPur–Ni NTA (ThermoFisher Scientific, #88226) columns for 30 min at 4°C for BG4 purification. Wash buffer (20 mM Tris–HCl pH 8, 500 mM NaCl, 20 mM imidazole, protease inhibitors) was used to perform two washes and BG4 antibody was finally eluted using 5 ml elution buffer (20 mM Tris–HCl pH 8, 250 mM NaCl, 250 mM imidazole). Eluted fractions containing BG4 were pooled together and dialysed in PBS + 20% glycerol overnight at 4°C. BG4 was then concentrated using Amicon 3K (ThermoFisher Scientific, #88525) and aliquots were stored at –20°C.

### iMab pull-down and western-blot

Biotinylated oligonucleotides (Supplementary Table S2) were resuspended in 100 mM phosphate buffer at 1.5 μM final concentration, heat-denatured at 95°C for 5 min and cooled to room temperature overnight. Next, folded oligonucleotides were immobilized on streptavidin-coated magnetic beads (Dynabeads™ M-280 Streptavidin, ThermoFisher Scientific, #11205D) and incubated with FLAG-tagged iMab (Absolute antibody, #Ab01462-30.135) 100 ng/sample for 1 h in ice bath. Excess antibody was washed four times with 50 mM Tris–HCl pH 7.5-150 mM NaCl and once with PBS. Western blot was performed according to previously reported procedures (10). Briefly, beads were resuspended in PBS, denatured and the supernatants were loaded on a 12% SDS-PAGE. The gel was then transferred on a PVDF membrane, blocked in 2.5% PBS-milk buffer, incubated with the anti-FLAG antibody 1:1000 (Sigma Aldrich, #F3165), washed in 0.1% PBS–tween, and incubated with secondary goat anti-mouse 1:4000 (Merck-Millipore #12-349) HRP antibodies. Images were acquired on the Alliance Uvitec (Uvitec Ltd. Cambridge, Cambridge, United Kingdom) instrument by HRP bioluminescence measurement. Experiments were performed in duplicate.

### CUT&Tag

The CUT&Tag protocol was adapted from Kaya-Okur *et al.* (26) and Lyu *et al.* (11). Briefly, fresh WDLPS and HEK293T were harvested (300000 cells/sample), washed with wash buffer (20 mM HEPES pH 7.5, 150 mM NaCl, 0.5 mM spermidine, complete Protease Inhibitor EDTA-Free), and immobilized to concanavalin A-coated magnetic beads (Bangs Laboratories, #BP531) for 20 min at RT on an end-over-end rotator. The bead-bound cells were incubated with antibody buffer (wash buffer supplemented with 1% BSA, 2 mM EDTA and 0.05% digitonin) overnight at 4°C on a nutator with the following primary antibodies: anti-H3K4me3 (1:50 dilution, rabbit monoclonal, Cell Signaling Technology, #C42D8), FLAG-tagged iMab (4 μg) and FLAG-tagged BG4 (500 ng). BG4 and iMab antibody-incubated cells were then subjected to mouse anti-FLAG antibody incubation at RT for 1 h on the nutator (1:100 dilution) and subsequently incubated with secondary antibodies. Rabbit anti-mouse IgG and guinea pig anti-rabbit IgG secondary antibodies were diluted 1:100 in dig-wash buffer and samples were nutated at RT for 1 h. After three washes with 800 μl dig-wash buffer, beads-bound cells were resuspended in dig-300 buffer (20 mM HEPES pH 7.5, 300 mM NaCl and 0.5 mM spermidine, 1% BSA and 0.01% digitonin) with 1:20 dilution of pA-Tn5 adapter complex (CUTANA™ pAG-Tn5 for CUT&Tag, EpiCypher, #15–1017) and nutated at RT for 1 h. Beads were washed with 800 μl dig-300 buffer three times, followed by tagmentation in 200 μl of tagmentation buffer (Dig-300 wash buffer with 10 mM MgCl_2_) at 37°C for 1 h. To stop tagmentation and digest proteins, 15 mM EDTA, 0.1% SDS and 500 mg/ml proteinase K were added and further incubated at 63°C for another 1 h. Supernatants containing DNA fragments were purified by phenol-chloroform extraction using phase-lock tubes (Qiagen MaXtract High Density, Qiagen, #129046) and libraries were amplified using NEBNExt HiFi 2× PCR master mix with uniquely barcoded i5 and i7 primers (29). The clean-up was performed with Agencourt AMPure XP beads (Beckman Coulter, #A63881) and the obtained libraries were analyzed with Bioanalyzer (Agilent) to evaluate the size distribution. Samples were sequenced paired-end on an Illumina NextSeq500 platform using 38 bp reads.

### Mapping pipeline

Sequencing data were uploaded to the Galaxy web platform and the public server at usegalaxy.org, together with R (version 4.2.1 – RStudio, https://www.R-project.org/, version 2022.07.0), were used to analyze all data (30). After quality control with FastQC (http://www.bioinformatics.babraham.ac.uk/projects/fastqc), reads were aligned to the human reference genome (GRCh38) using Bowtie2 (v2.4.2) (31) with the following paired-end options: -I 10 -X 700 –fr hg38 –very-sensitive-local. Normalized (RPGC, 1× Genome Coverage) bedgraph tracks were generated using deepTools (v3.5.1) bamCoverage (32) with binSize 5 and the respective genome size. iM- and G4-peaks were called with SEACR (v1.3) (33) in stringent mode with 0.01 as threshold and high confidence peaks among three biological replicates were obtained with BEDTools intersect (34). Annotation of peaks was performed with the R package ‘ChIPseeker’ (35) and Venn diagrams were plotted with R package ‘VennDiagram’ (36). G4-iM shared and unique datasets were obtained with BEDTools intersect function (34). For GVIZ (37) visualization of genomic regions, bigwig files of all biological replicates were merged using deepTools2 (32) BigwigCompare function and scaled referring to the sample with the lowest sequencing depth. Bar, donut, and pie charts were obtained with GraphPad Prism (version 9 for Windows, GraphPad Software, San Diego, California USA, www.graphpad.com). Fraction of reads in peaks (FRiP) was calculated using deepTools module CountReadsPerBin by sampling the genome into 10000 positions of size 1 base (32). Saturation curves were generated by random sampling of the original files with seqtk (https://github.com/lh3/seqtk) to cover varying sequencing depth, to obtain downsampled bam files. The downsampled files were processed as above up to SEACR peak calling, then the mean read number derived from the three biological replicates, falling within the called peaks were estimated. Library complexity was calculated with the preseq program (38) using *c_curve* functionality. Public R-loop CUT&Tag data were analyzed accordingly, while ATAC-seq data were analyzed as previously reported (39). For both R-loop CUT&Tag and ATAC-seq, peaks overlapping with iMab-/BG4-CUT&Tag results were identified with BEDTools intersect (34).

### Heatmaps

All heatmap plots were generated using deepTools suite (32). Refseq validated gene coordinates (hg38) were recovered from https://www.genome.ucsc.edu/cgi-bin/hgTables/ and used to display reads occupancy at the TSS. Bin of 25 bp were used to average the score over the region length. Bigwig files used to compute *z*-score were library size scaled. When biological replicates were not plotted individually, a mean bigwig file computed by means of WiggleTools was employed (40).

### G4- and iM-forming sequences analysis

DNA sequence of high confidence peaks was extracted with ‘write2FASTA’ function of R package ‘ChIPpeakAnno’ (41) and G4/iM sequence motifs were searched with MEME-ChIP online tool (v5.5.0) (https://meme-suite.org/meme/tools/meme-chip). For G4/iM prediction, FASTA files were used as input for Quadparser tool analysis. Quadparser script was obtained from https://github.com/dariober/ as indicated previously (42) and applied with three different regular expressions, which indicate different stringency levels: low stringency, four G-tracts with at least two Gs each; medium stringency, four G-tracts with at least three Gs each; high stringency, five G-tracts with at least three Gs each. For each searching motif, two loop lengths were evaluated, 0–7 and 0–12. The fold enrichment plot was obtained by dividing the actual counts for each prediction group within peak regions, by counts of the same regions upon random reshuffling using the BEDTools shuffle command. Results are reported as the average of 10 randomizations.

### Circular dichroism

Oligonucleotides (Supplementary Table S2) were diluted to final concentration of 3 μM in 20 mM phosphate buffer, 80 mM KCl over a pH range of 5.4–7.4. Where indicated, PEG200 (Sigma-Aldrich, #88440) was added at 40% (v/v) final concentration. Samples were heated at 95°C for 5 min and then slowly cooled to room temperature overnight. CD spectra were recorded on a Chirascan-Plus equipped with a Peltier temperature controller using a quartz cell with a 5 mm optical-path length. CD data were measured at 20°C from wavelength 230 to 320 nm. Acquired spectra were baseline-corrected for signal contribution from the buffer, and the observed ellipticities were converted to mean residue ellipticity according to θ = degree × cm^2^ × dmol^−1^ (molar ellipticity). CD spectra were performed in duplicate and plotted with R.

### RNA-seq analysis

Total RNA was extracted from HEK293T (3 million cells per sample) using the GeneJET RNA Purification Kit (Thermo Fisher Scientific, #K0731) according to the manufacturer's instructions. RNA concentration and quality were checked using the Qubit (Thermo Fisher Scientific) and the TapeStation System (Agilent), respectively, before and after library construction. RNA-seq libraries were generated using Quant Seq 3′ mRNA-seq Library Prep kit (Lexogen). Sequencing was performed on NextSeq500 ILLUMINA instrument to produce at least 35 million reads (75bp SE) per sample. The experiment was performed in three independent biological replicates. Sequencing data were uploaded to the Galaxy web platform and the public server at usegalaxy.org, together with R (version 3.4), were used to analyze all data. RNA-seq data for the WDLPS cell line were downloaded from GEO (accession number: GSE145543) (10). Bioinformatic analysis on obtained reads for both cell lines was performed as previously reported (10).

### Reactome pathway enrichment analysis

Reactome (43) pathway enrichment analysis was performed using as input those genes whose promoter holds iM-structures previously identified with R package ‘Chipseeker’ (35) annotation to the promoter within ±1 kb distance. This analysis was performed for top HEK293T iM-peaks with default settings. Reactome database is available at https://reactome.org/.

## RESULTS

### Genome-wide mapping of native iMs by iMab-CUT&Tag

**Figure 2.**
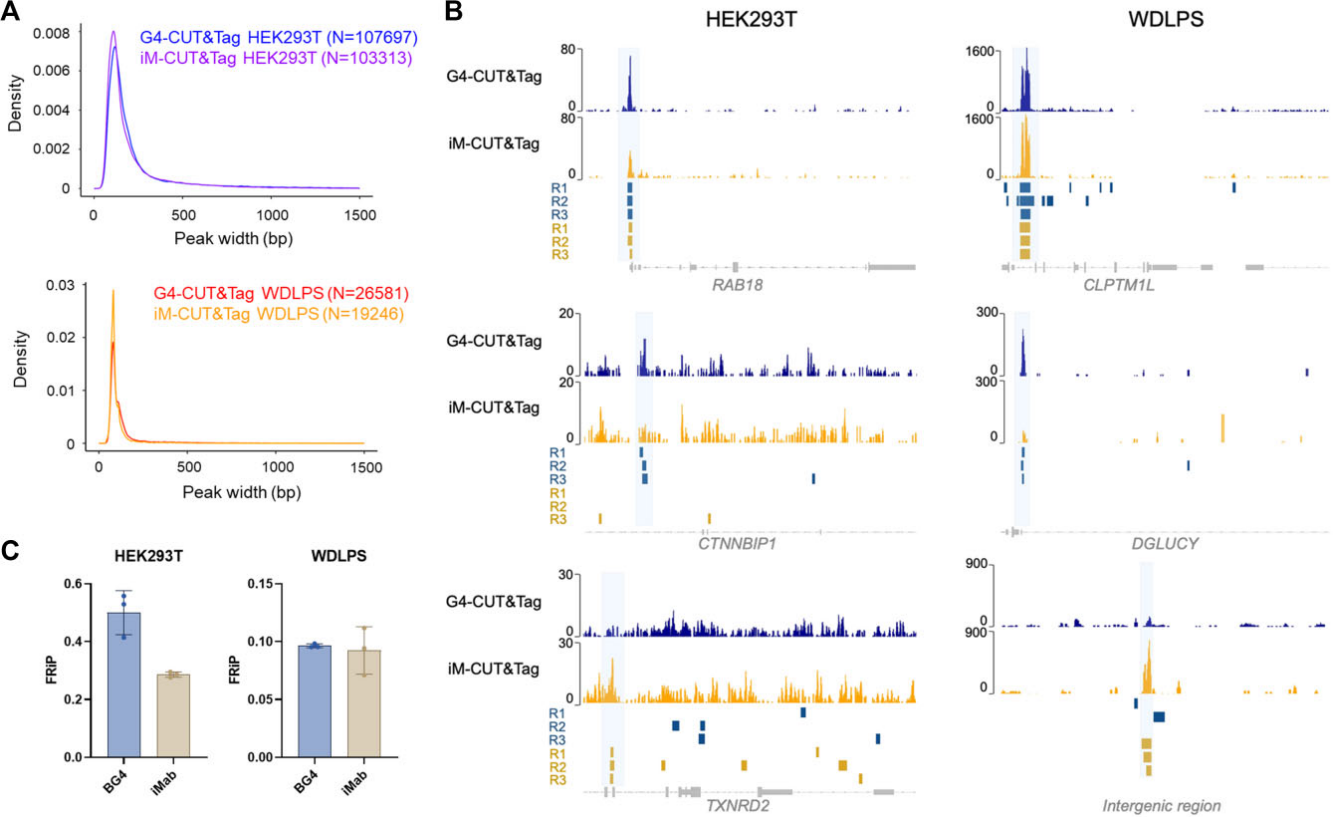
iM- and G4-CUT&Tag efficiency among replicates. (**A**) Peak width distribution; top: HEK293T iM/G4-CUT&Tag samples; bottom: WDLPS iM/G4-CUT&Tag samples. (**B**) Visualization of representative genomic regions of iM- (yellow) and G4- (blue) CUT&Tag tracks in HEK293T (left) and WDLPS (right) cell lines. Reads were aligned to the human genome (hg38) and normalized by reads per million. SEACR-identified peaks for the three biological replicates (R) are shown below each peak as colored boxes (yellow for iM and blue for G4 samples). Gene annotation, when available, is reported at the bottom of the track. Representative peaks present in both iM- and G4-CUT&Tag samples (top), or unique peaks present in only one of the two sets are shown (middle and bottom). (**C**) FRiP generated for iM- and G4-CUT&Tag samples from both HEK293T and WDLPS cell lines. Error bars represent standard deviations of three independent replicates.

To study the distribution of iMs in the genome of living cells, we used iMab, the available anti-iM antibody (22), with iM-CUT&Tag. BG4, the most widely used anti-G4 antibody (5), was simultaneously used in the G4-CUT&Tag protocol (11), to also map G4 motifs. The binding selectivity of iMab in our conditions was first assessed by pull-down analysis coupled with Western blot (Supplementary Figure S1A). We used four iM-, two G4-forming sequences (44,45) and two unstructured DNA sequences as controls (Supplementary Table S2), as assessed by CD analysis (Supplementary Figure S1B). We found that iMab was totally selective for iMs. With CUT&Tag, we compared iM and G4 formation at the genomic level in two different cell lines: an embryonic epithelial-like line (HEK293T) and a tumor fibroblast-like line (WDLPS) derived from the well-differentiated liposarcoma. Briefly, as depicted in Figure 1D, mildly permeabilized cells were incubated with the antibodies of interest and, following enzymatic cleavage and tagmentation, the immunoprecipitated DNA fragments were subjected to next-generation sequencing. For each CUT&Tag reaction, three biological replicates were analyzed separately up to SEACR peak calling (33). Thousands of peaks were identified for each tested condition (Supplementary Table S1). The reaction efficiency was corroborated calculating saturation curves: all CUT&Tag reactions approached saturation, also in library complexity analysis (38), and populated peaks more rapidly at low sequencing depths (Supplementary Figure S1C and D). Similar trends were observed when published data were analyzed with our pipeline (Supplementary Figure S1C). The consistency of the biological replicates was measured by Pearson's correlation, which yielded high coefficients, hence indicating high reproducibility in both cell lines and with both tested antibodies (Supplementary Figure S2A). Density heatmaps among replicates showed consistent distribution of reads, both when centered on the called peak regions (Supplementary Figure S3A, B) and at the TSS of all human genes (Supplementary Figure S3C). Sample reproducibility was visually inspected and further confirmed by GVIZ tracks, both at shared peaks identified with anti-iM and anti-G4 antibodies and at peaks recovered with only one of the two antibodies (Figure 2B). Peak size distribution analysis showed that the median peak size in all samples was ∼150 bp, which is in accordance with previous CUT&Tag data (Figure 2A) (46). Both iM- and G4-CUT&Tag-derived peaks in both cell lines were significantly enriched in GC content, with respect to the whole genome GC abundance (47) (*P*-value < 0.0001) (Supplementary Figure S2B), and they reported FRiP values in line with the literature (Figure 2C, Supplementary Figure S1C) (11,48). Taken together, these data fully support our iM-CUT&Tag method as an efficient iM-mapping approach.

### iM-CUT&Tag efficiently provided iM-folding sequences enriched at promoters

**Figure 3.**
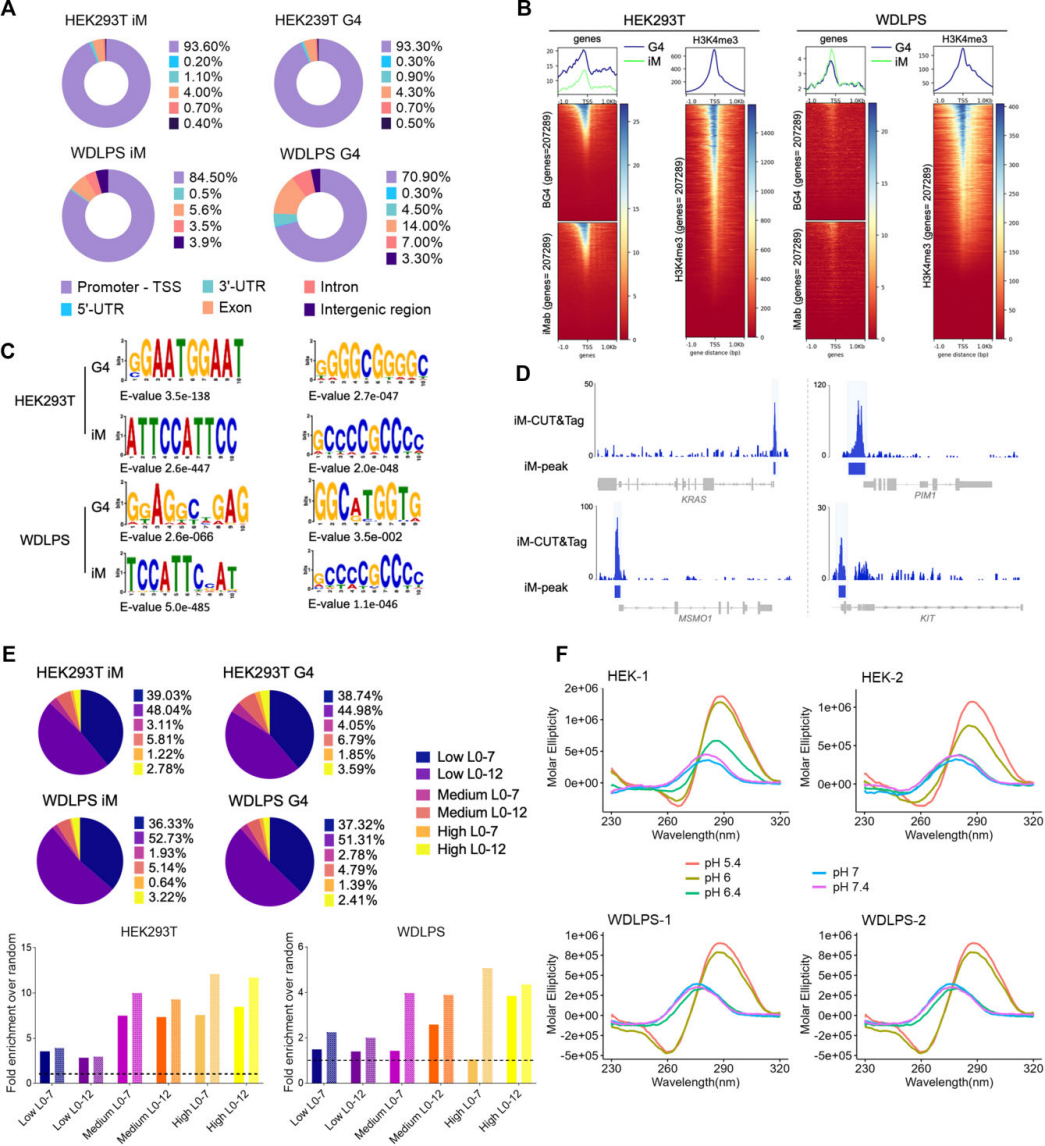
iM- and G4-CUT&Tag sequencing results. (**A**) Donut charts representing the distribution of high-confidence peaks for both iM- and G4-samples from HEK293T (upper charts) and WDLPS (lower graphs) in functional genomic regions, according to ChIPseeker annotation. The percentages are normalized over the genomic abundance of each functional region and numeric values are reported in the legend for each sample. (**B**) Average plot (top) and density heatmaps (bottom) of G4- and iM-CUT&Tag peaks in HEK293T (left) and WDLPS (right). For each cell line, peak density is referred to the TSS within ±1 kb distance, and the corresponding H3K4me3-CUT&Tag density profile is shown on the right. (**C**) Investigation of recurrent motifs research using MEME-ChIP from the high confidence iM- and G4-CUT&Tag peaks. For each sample, two motifs with their *E*-value are reported. (**D**) Visualization tracks of iM-CUT&Tag profiles, referred to known iM-forming sequences *in vitro*. Reads were aligned to the human genome hg38 and normalized to reads per million. SEACR-identified peaks are shown as colored boxes below each peak. Gene annotation is reported at the bottom of each track. (**E**) Percentages of putative iM- and G4-forming sequences normalized on total peaks (pie charts) and fold enrichment (bar chart), relative to randomly composed sequences (average of 10 randomizations per sequence) in HEK293T (top) and WDLPS (bottom). Plain bars indicate iM-forming sequences, pattern-filled bars indicate G4-forming sequences. iM/G4 prediction was performed for the following motifs: *low stringency*, four C/G-tracts with at least two Cs/Gs each; *medium stringency*, four C/G-tracts with at least three Cs/Gs each; *high stringency*, five C/G-tracts with at least three Cs/Gs each. For each searching motif, two loop sizes, short (0–7) and long (0–12), were evaluated. The dashed line indicates fold enrichment = 1. (**F**) CD spectroscopy analysis of representative identified CC-, CCC- and CCCC-tract piMs in HEK293T (top) and WDLPS (bottom). Oligonucleotides were folded in phosphate buffer and analyzed at different pH levels, as indicated.

High-confidence datasets were obtained by intersecting peaks from the three biological replicates of each sample (23902 iM- and 29640 G4-peaks in HEK293T; 860 iM- and 3400 G4-peaks in WDLPS). These represent up to 65% of all peaks, which is in line with previously reported G4-CUT&Tag data (48). In the same cells, 80–90% overlapping was identified with our positive CUT&Tag control histone mark H3K4me3. The iM/G4 regions shared among biological replicates represent the most robust fraction of quadruplex structures within the dynamic iM/G4-landscape in the cell context; we thus performed all subsequent analyses on the high confidence datasets. We found that almost all iM-peaks were annotated at gene promoters in both cell lines (Figure 3A). For this first analysis, we also checked the iM-peaks present in each of the three replicates individually and confirmed that they were also predominantly annotated at the promoter (Supplementary Figure S4), validating the non-random distribution of the iMs found. G4-peaks were also mostly annotated at promoters confirming previous data (Figure 3A) (10,11). Both iM- and G4-peaks in both cell lines showed the highest density within 1 kb upstream of the TSS (Figure 3B) and colocalized with the regions marked by H3K4me3, a histone modification associated with open chromatin and active transcription sites (Supplementary Figure S5) (26), suggesting that not only G4s (9,10), but also iMs may be associated with gene transcription regulation. MEME-ChIP motif search validated the specificity of the iM- and G4-CUT&Tag analyses providing C/G-rich sequences as enriched motifs with highly significant E-values. In particular, the SP1 binding motif, previously reported to be enriched in G4 sites (13), was retrieved in both iM- and G4-peaks in HEK293T; the centromeric repeat ‘CCATT’, previously reported to fold into iMs (1), was the most enriched motif in both cell lines (Figure 3C). Visual inspection of the iM-peaks further confirmed the efficiency of the iM-CUT&Tag analysis, which detected several sequences, i.e. *KRAS*, *MSMO1*, *PIM1* and *KIT* promoter regions, previously reported to fold into iMs *in vitro* (Figure 3D) (17,20,49).

To confirm iM folding within the identified iM-peaks, we performed bioinformatic prediction analysis searching for putative iM-forming sequences (piMs) consisting of i) C-tracts with 2 or more Cs and ii) short (0–7) or long (0–12) loops. We defined stringency levels based on C-tract length and number of repeats (low = four tracts with 2Cs, medium = four tracts with 3 Cs, high = five tracts with 3 Cs) (Figure 3E). A fifth ‘spare tyre’ C/G-tract was considered, as this arrangement has been shown to be abundant at G4s in most of the characterized oncogene promoters (50). For each searched motif, the fold enrichment was estimated by comparison with random reshuffling across the genome. We observed that all the structures searched for were likely to be present within the peaks (fold change > 1) and, very encouragingly, that the enrichment increased with the increasing prediction stringency (Figure 3E). Sequences from HEK293T showed higher enrichment than those from WDLPS. To validate the ability of the identified piMs to actually fold into iMs, we performed circular dichroism (CD) analysis of four representative sequences from both cell lines (Figure 3F and Supplementary Figure S6). Notably, iM-peaks identified sequences consisting of C-tracts of different length, i.e. formed by 2, 3 or 4 Cs (Supplementary Table S2). The C-tract length has been reported to be proportional to iM stability, with the longer the better (1). In contrast, our CD analysis showed that all tested sequences exhibited iM CD spectra at acidic pH, with characteristic iM profile (maximum peak λ∼285 nm, minimum peak λ∼260 nm) (51) regardless of the C-tract length (Figure 3F, Supplementary Figure S6). The folding propensity of these sequences was pH-dependent, as expected in *in vitro* analysis, considering that folding at neutral pH requires longer C-tracts (17,52). For all the sequences tested, including those with the shortest C-tracts, i.e. with 2Cs, iM folding was optimal at pH 5.4. Within the iM-CUT&Tag peaks, we identified previously characterized sequences, such as the KRAS and the KIT iMs (Figure 3D), which also do not fold at physiological pH *in vitro* (20,49). We next tested whether the crowding/dehydrating agent PEG200 improved iM stability at physiological pH: we observed a general bathochromic shift not sufficiently broad to provide full iM spectra (Supplementary Figure S6C, D). These data suggest that in cell conditions favor iM formation, even at sequences with short C-tracts that were previously considered too unstable (52); they also indicate that *in vitro* conditions cannot fully recapitulate the cell environment.

### iMs and G4s are distinctive features of each cell type

**Figure 4.**
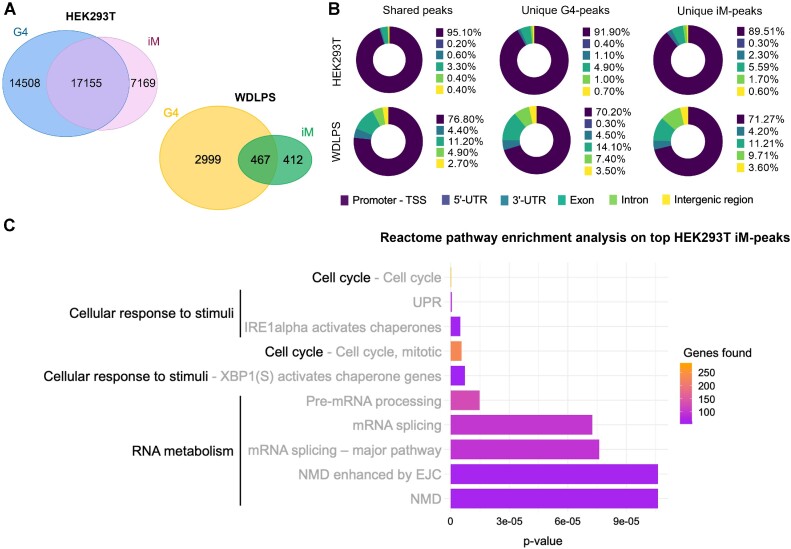
iM and G4s distribution and possible biological role. (**A**) Venn diagrams showing shared and unique peaks from iM- and G4-CUT&Tag analyses in HEK293T (left) and WDLPS (right). (**B**) Donut plots showing the distribution of shared and unique peaks in HEK293T (top) and WDLPS (bottom) in functional genomic regions, according to ChIPseeker annotation. Percentages are normalized over the genomic abundance of each functional region, and numerical values are reported in the legend for each sample. (**C**) Reactome pathway enrichment analysis of top iM-peaks annotated to TSS from HEK293T cells. The top 10 most over-represented pathways are listed by *P*-value and their colors indicate the number of genes found. *P*-value < 0.001. UPR: Unfolded Protein Response; NMD: Nonsense-Mediated Decay; EJC: Exon Junction Complex.

The data collected so far prove that iMs form in live cells, along with G4s. Because of the different distribution of iMs and G4s in HEK293T and WDLPS cells, we wondered whether they might form in the same genomic regions. To this end, for each cell line we identified shared peaks, i.e. common peaks among iM- and G4-CUT&Tag analyses. In HEK293T cells, we counted 17155 shared peaks, while the remaining unique iM- and G4-peaks were moderately fewer (Figure 4A). In WDLPS cells, we found 467 shared peaks, representing 54% of all iM-peaks (Figure 4A). In contrast, the much larger G4 dataset contained 2999 unique G4-peaks (Figure 4A). In HEK293T cells, the three groups (shared, unique G4- and unique iM-peaks) were almost entirely located at gene promoters (Figure 4B). In WDLPS cells, slightly lower percentages were found, especially for the unique iM- and G4-peaks, where ∼10% of both were located at exons (Figure 4B). These data indicate that iMs and G4s can fold independently in cells (unique peaks); as for the shared peaks, it is not possible with this technique to define whether iMs and G4s are folded in the exact same DNA sequence in the same cell or in the same genomic region in different cells.

To better investigate this issue, we classified iM- and G4-peaks (both unique and shared with the counterpart) into bottom, middle and top SEACR signal intensity ranges, according to quartile division (bottom ≤ 1st quartile, middle = interquartile range, top ≥ 3rd quartile) (Supplementary Tables S3 and S4). For the iM-peaks, we observed that the majority of the peaks in the top signal range belonged to the shared peak datasets (5294/5310 in HEK293T; 170/192 in WDLPS). The middle signal range peaks, which represented the most abundant subset in both cell lines, were also mostly present in the shared peak datasets (77% in HEK293T; 58% in WDLPS). The bottom signal range peaks showed the lowest distribution in the shared peaks (37% and 44% in HEK293T and WDLPS cells, respectively). This distribution among signal ranges was confirmed for G4-peaks in HEK293T, but not in the cancer cell line, where the majority of G4s were not only in the unique dataset, but also in the top signal range (433/728) (Supplementary Table S4). Overall, these data suggest that the contextual formation of the iMs and G4s in the same genomic region may be favored, and also indicate that the iM- and G4-landscapes are cell line-specific. To gain insight into the possible biological role of iMs and G4s present in the same genomic region, we performed Reactome pathway enrichment analysis considering the top iM-peaks (Supplementary Table S3) from HEK293T, of which 99% are present in the shared dataset. Genes with most robust iMs mainly belonged to pathways related to the cell cycle, cellular response to stimuli and RNA metabolism (Figure 4C). These data corroborate the involvement of alternative DNA secondary structures in key cellular pathways.

### iMs are associated with transcription and open chromatin

**Figure 5.**
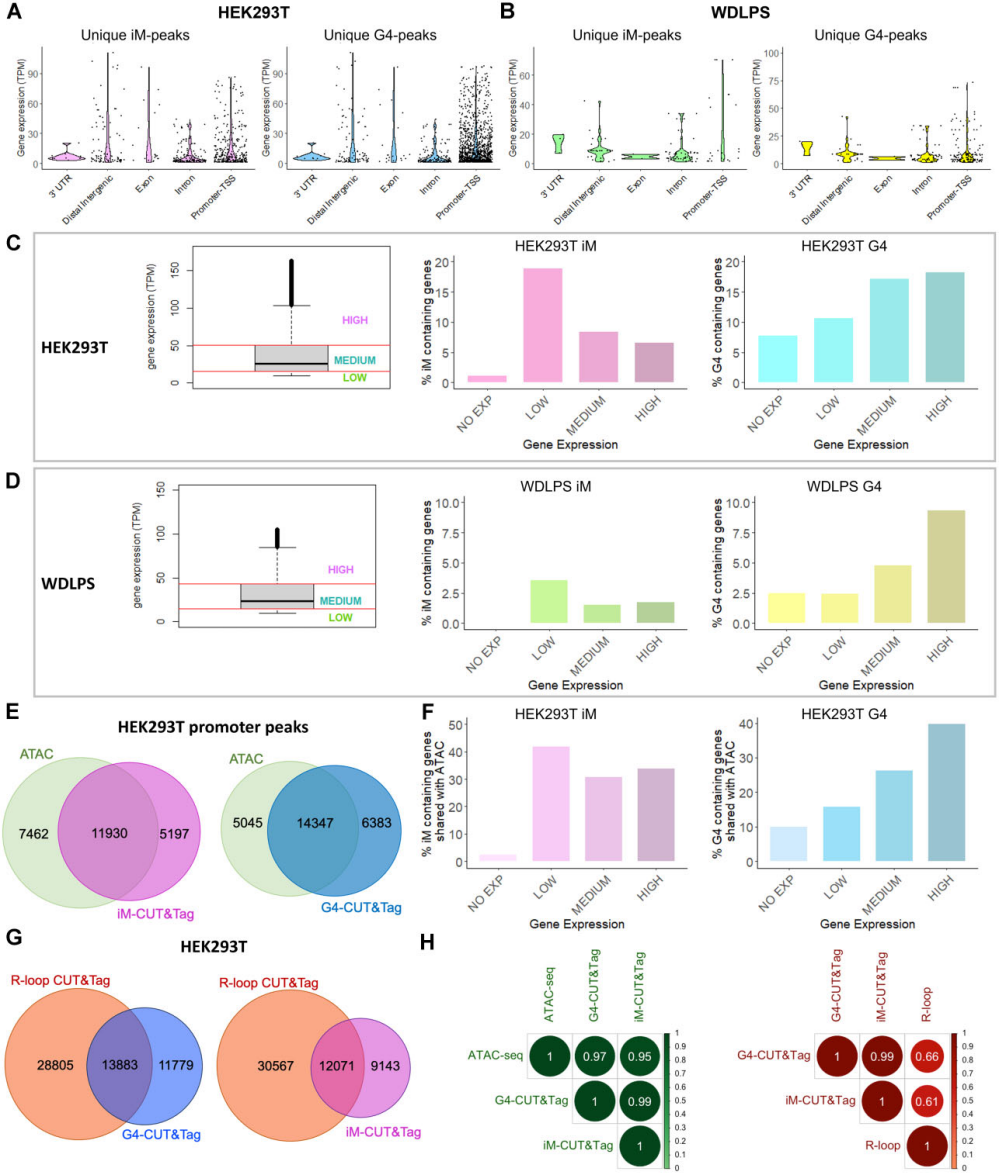
iMs association with transcription, open chromatin regions and DNA-RNA hybrid structures. A-B) RNA-seq integration data: gene expression distribution was reported in transcripts per million (TPM) of genes included in iM-unique peaks (right panel) and G4-unique peaks (left panel) from HEK293T (**A**) and WDLPS (**B**). Genes were grouped according to the functional annotation of the immunoprecipitated region. Outliers were excluded with the interquartile range method. C-D) Gene expression distribution of all the expressed genes (at least one transcript per gene) in HEK293T (**C**) and WDLPS (**D**) cells according to RNA-seq data. Three expression categories were defined based on the quartiles of expression distribution: the first quartile corresponds to low expression, the central two quartiles to medium expression and the upper quartile to high expression. The percentage of iM- and G4-containing genes is shown according to their expression level (no, low, medium, or high expression). (**E**) Venn diagrams showing shared peaks between ATAC-seq (green) and iM- (purple) and G4-CUT&Tag reactions (blue). Peak subsets were defined according to ChIPseeker annotation to promoter regions. (**F**) Percentage of iM- (pink) and G4-containing (blue) genes in HEK293T shared with the ATAC-seq dataset, according to their expression level (no, low, medium, or high expression). (**G**) Venn diagrams representing the intersection between R-loop CUT&Tag (orange) and CUT&Tag data (G4-CUT&Tag in blue and iM-CUT&Tag in purple). (**H**) Correlation plots of HEK293T CUT&Tag versus ATAC-seq (left) and versus R-loop CUT&Tag (right). Pearson's correlation values for all samples are reported.

Our data indicate that both iMs and G4s are predominantly present in regulatory genomic regions, suggesting that both structures may be involved in transcription regulation. To investigate this further, we integrated CUT&Tag data with RNA-seq analysis to correlate secondary structure formation with gene expression. We found that genes containing both iMs and G4s in the promoter region within 3 kb from the TSS produced the highest number of transcripts in both cell lines (Figure 5A, B). We observed a strong correlation between G4 enrichment and the most highly expressed genes in both cell lines tested (Figure 5C, D), as expected (10,11,13). iMs were also associated with increased gene expression but, surprisingly, mainly in the genes with the lowest expression level (Figure 5C, D), suggesting that, despite a similar distribution at gene promoters (Figure 4B), iMs and G4s may regulate transcription through different mechanisms.

Previous studies have shown that G4 formation depends more on chromatin accessibility and remodeling, rather than on transcriptional activity. Subsequently, folded G4s in open chromatin regions were shown to contribute to the recruitment of various transcription factors, thereby increasing transcript levels (53).

For iMs, the relationship with transcription and open chromatin has not been investigated. Therefore, we combined iM- and G4-CUT&Tag data in the HEK293T cells with available public ATAC-seq data in the same cell line. Correlation analysis revealed high Pearson's correlation values for both G4- and iM-CUT&Tag samples (Figure 5H). Intersection of the identified peaks showed that 60% of all iM- and G4-CUT&Tag peaks (Supplementary Figure S7A) were located in open chromatin regions. Up to 84% of ATAC-positive regions were located at gene promoters (Supplementary Figure S7B) and 70% of both promoter iM- and G4-CUT&Tag peaks (Figure 5E) were in open chromatin. Thus, as already shown for G4s (53), iMs may also be influenced by the chromatin state. However, 30% of iM/G4-CUT&Tag peaks were not shared with ATAC-peaks, suggesting that open chromatin may not be the only driving force for iM/G4 formation. These data also exclude a technical bias of the iM/G4-CUT&Tag analysis for open chromatin regions (54). Integration with RNA-seq data of CUT&Tag and ATAC shared peaks confirmed the association of iMs with transcripts that have the lowest expression level, in contrast to G4s (Figure 5F).

Next, we investigated the overlap of iMs with DNA-RNA hybrids (R-loop), whose relationship with G4s has been previously reported (11,55,56). We combined deposited R-loop CUT&Tag data from HEK293T cells (57) with both iM- and G4-CUT&Tag results. A positive correlation with both G4s (r = 0.66) and iMs (r = 0.61) was observed (Figure 5H). iMs shared more than 50% of the peaks with R-loop CUT&Tag, similar to G4s (iMs: 57%; G4s: 54%) (Figure 5G) (11).

## DISCUSSION

Here, we investigated and compared the iM- and G4-landscape in two live cell lines. To this end, we optimized and successfully performed CUT&Tag sequencing using iMab, the available anti-iM antibody (further confirmed here to selectively bind iMs, Supplementary Figure S1A) (22), together with BG4, the most widely used anti-G4 antibody (5), as previously reported (9–11). This method provides a new approach for the genome-wide mapping of iMs (and G4s), the folding of which is highly dynamic in cells (9,10). In this context, we observed less peak overlap among iM and G4 biological replicates than for the histone mark replicates. Indeed, the abundance and genome specificity of the latter make them suitable positive controls for the CUT&Tag protocol (58), while we ascribe the lower iM/G4 replicate overlapping to the dynamism of iMs and G4s in live cells.

We found that both iMs and G4s are mainly, but not exclusively, located in open chromatin regions, mostly at gene promoters upstream of the TSS. These results are consistent with previously reported data from different techniques, including experiments with fixed or fragmented cells (23,24,59), in which iMs were mostly folded during the G1 phase of the cell cycle, when transcription is active, whereas G4s were more abundant during DNA replication (S phase) (59), as both processes occur in open chromatin regions (60). However, a different association with transcription was obtained for iMs and G4s: while G4-containing genes were confirmed to be related to highly enhanced transcription, iMs displayed a different behavior, with most iM-containing genes showing expression levels in the lowest range. These results suggest that iM and G4 folding may be physically correlated, but that their formation has functionally different effects.

In addition, the identification of folded quadruplexes in condensed chromatin (Figure S7A) provides evidence that both iMs and G4s are associated with the regulation of different pathways other than transcription, expanding their biological relevance. The Balasubramanian group has previously shown that transcription itself is not a primary determinant of G4 folding at promoters (53): ChIP-seq analysis performed after chemical inhibition of both transcription elongation and initiation revealed no changes in G4s enrichment, indicating that G4s are formed independently of transcription. Considering the common distribution between iMs and G4s, it is tempting to speculate that iMs may behave accordingly.

How iMs/G4s form in condensed chromatin would need further mechanistic studies; the chromatin condensation level relies on epigenetics modifications (61) and in turn epigenetics have been reported to affect iM and G4 formation (62–65). Hence, it is possible that iMs/G4s form in more relaxed, yet still condensed chromatin and possibly contribute to its regulation.

However, the predominant presence of both structures in regulatory regions, such as promoters, may indicate a specific association with active transcription. Indeed, we found that approximately half of the iM/G4-peaks overlapped with R-loops. As R-loop formation implies an unfolded nature of the DNA, they have been related to G4s, which in turn have been shown to influence R-loop formation and stability, and consequently transcription (4,66-67). Considering that the top HEK293T-CUT&Tag peaks contain both iMs and G4s, and that the two structures were correlated with different gene expression levels, we propose that their concomitant presence is a means to finely tune transcriptional regulation.

The acidic requirement for iM folding is still a matter of debate since it questions their biological formation and relevance *in vivo*. Here, iMab-immunoprecipitated genomic regions were remarkably enriched in C-rich sequences that also fold into iMs *in vitro*, as demonstrated by CD analysis, with a clear pH-dependency. Encouragingly, several regions that had been previously reported to fold into iMs *in vitro* with different transitional pH (pH_T_), such as *KRAS* (pH_T_ 6.2–6.9) (20), *MSMO1* (pH_T_ 6.7) (17), *PIM1* (pH_T_ 7) (17) and *KIT* (pH_T_ 5.8–6) (49), were also present in our iM dataset. This evidence suggests that the cellular environment plays a crucial role in determining iM folding. To this end we performed CD analysis in crowding conditions using polyethylene glycol (68), which limitedly improved iM spectra at physiological pH (Supplementary Figure S6C), therefore confirming that i) additional cell factors likely participate in iM folding; ii) *in vitro* testing conditions do not properly reflect the cellular context, even when crowding agents are used. Moreover, we found iM-peaks to be enriched in CC-tract iMs: 2-layered iMs have been mainly ignored so far, as they were considered too weak to fold, except for one sequence that folds *in vitro* at pH ∼ 5 (17). This new information indicates that many more regions than previously thought can fold in iMs in cells, greatly expanding their relevance within genomes. We also observed that many sequences contained an additional C-tract, which has been defined as a ‘spare tyre’ in G4s (50): this arrangement, which allows alternative conformational folding and possibly alternative binding partners, is well represented in the human genome for both iMs and G4s. Taken together, these data strongly suggest that iM folding in cells is not only triggered by acidic pH, but mainly by additional conditions present in live cells, such as binding proteins and cell crowding agents, as previously hypothesized (18,21).

Since iMs and G4s can in principle form in complementary sequences, a long-debated question is whether formation of the two structures occurs at the same site and whether it is interdependent. The CUT&Tag technique used here cannot determine whether complementary genomic regions can fold into both quadruplex structures, since the detection limit size is 150 bp, i.e. the size of the immunoprecipitated sequences, where typically multiple G4s/iMs can form on the same and complementary strands. However, our analysis clearly indicates that iMs and G4s do form independently, as only one of the two structures was found in several immunoprecipitated sequences (unique peaks).

At the same time, the iM sequences identified with the highest degree of confidence (the highest SEACR signal intensity) were always found in both iM and G4 pools (shared peaks) (Supplementary Table S3). These data indicate that the contextual folding of quadruplexes in the same genomic region could be favored in open chromatin regions, considering the high overlap with ATAC-seq data (Figure 5E). In this scenario, iMs and G4s do not necessarily fold at complementary sequences, but close by, within the approximately 150 bp of the identified sequences, which can indeed host multiple iMs/G4s at different sites. As for the top G4-sequences, this trend was not confirmed for the WDLPS cell line, indicating that iM- and G4-landscapes are to be considered cell specific features. In fact, in the cancer cell line we reported a higher G4 enrichment and observed that 60% of the top G4-peaks folded independently from iMs. These data suggest that, despite the similar genomic distribution, G4s are functionally more relevant than iMs in this cell line, and thus may be validated as hallmarks of cancer. However, it should be noted that we identified less G4-peaks in the WDLPS versus the HEK cell line. Although G4s have been generally considered to be more abundant in cancer cells, as they are mostly located in oncogene promoter regions (69), their relative enrichment is highly cell type-specific, as further confirmed by our data, thus limiting the relevance of comparative analysis between different cell lines. As for iMs, our data represent the first genome-wide investigation in the cellular context, showing that iMs are generally less abundant than G4s.

This new evidence of the existence of iMs and their interplay with G4s in cells could be exploited in the study of most human diseases, such as cancer, infections and neurodegenerative disorders, where G4s have already been reported to play a key role (44,69–72). To this end, ligands targeting G4s have been developed, one of which is currently in phase II clinical trials for cancer treatment (73). Unfortunately, the reported iM ligands can also bind G4s to varying degrees, thus making a clear assessment of their biological activity impossible (74,75).

Our findings, by revealing the similar yet unique properties of iMs and G4s within the human genome and their distinctive relevance within cell types, expand the field of quadruplex structures and stimulate research into their role in disease and their use as drug targets.

## Supplementary Material

gkad626_Supplemental_File

## Data Availability

All genomic data produced in the present project (HEK293T-CUT&Tag, WDLPS-CUT&Tag, and HEK293T-RNA-seq) have been deposited in the NCBI GEO database under accession number GSE220882. Public data were downloaded from GEO: GSE145543 (WDLPS RNA-seq) (10) and GSE156400 (HEK293T R-loop CUT&Tag) (57), GSE173103 (saturation curve) (11), GSE181373 (saturation curve) (48). HEK293T ATAC-seq public data were retrieved from Sequence Read Archive via BioProject PRJNA380283 or SRA SRP103230 (39). Reactome database (https://reactome.org/) was employed to calculate the significant pathway enrichment. The complete list of human genes annotated on the GRCh38-hg38 reference genome was retrieved from BioMart Ensembl database (http://www.ensembl.org/biomart/martview). All data are available from the authors upon request. Custom-made R scripts are available at https://doi.org/10.5281/zenodo.8177215 and upon request from the corresponding author. Quadparser script was downloaded from https://github.com/dariober/, as indicated by Puig Lombardi *et al.* (42).
