# Supplementary material for: Genome-wide mapping of i-motifs reveals their association with transcription regulation in live human cells
Source: Nucleic Acids Res. 2023 Aug 2;51(16):8309–21. doi: 10.1093/nar/gkad626 (PMC10484731; doi:10.1093/nar/gkad626)
Supplement: gkad626_Supplemental_File [file gkad626_supplemental_file.pdf]

Supplementary Information

Genome-wide mapping of i-Motifs reveals their association with transcription regulation in live human cells

Irene Zanin[1], Emanuela Ruggiero[1], Giulia Nicoletto[1], Sara Lago[2], Ilaria Maurizio[1], Irene Gallina[1] and Sara N. Richter[1,3*]

[1]Department of Molecular Medicine, University of Padua, Padua, Italy

[2]Department of Cellular, Computational and Integrative Biology (CIBIO), University of Trento, Trento, Italy

[3]Microbiology and Virology Unit, Padua University Hospital, Padua, Italy

Content

Tables S1-S4 pp. S2-S4

Figures S1-S7 pp. S5-S10

Supplementary bibliography pp. S10

Table S1. SEACR peaks for all samples tested.

Cell line	Antibody	Replicate	Identified peaks
HEK293T	iMab	R1	41698
		R2	60804
		R3	53117
	BG4	R1	45551
		R2	57905
		R3	63981
WDLPS	iMab	R1	9031
		R2	5469
		R3	7263
	BG4	R1	12458
		R2	14153
		R3	8138

Table S2. Oligonucleotides used in the CD analysis.

C_n tracts (with n ≥ 2) are shown in bold. For each oligonucleotide, name and sequence are reported.

**Name**	**Sequence**
HEK-1	**CCCC**G**CCCC**G**CC**G**CCCC**TGTGT**CCCC**
HEK-2	**CC**G**CCC**GTCA**CCC**T**CC**
HEK-3	**CCCC**GCTGG**CCCC**G**CCCC**ATTT**CCCC**
HEK-4	**CCC**ACAGTA**CCC**AGG**CC**ACT**CC**CACAGCA**CCC**
WDLPS-1	**CC**TACACA**CC**TCA**CCC**T**CC**
WDLPS-2	**CCCCCC**TTAT**CC**TCAT**CCC**
WDLPS-3	**CC**G**CCC**GTCA**CCC**T**CC**
WDLPS-4	**CC**ATTCTTGT**CC**ATTCGATGATT**CC**ATTTGATT**CC**
iM_1	[Btn]-**CCCC**AGT**CCC**G**CCC**AGG**CC**ACG**CC**T**CCC**
iM_2	**CCC**TCT**CCC**TCT**CCC**TCT**CCC**TCT-[BtnTg]
iM_3	[Btn]-AAGCCACGTG**CCCC**ATGC**CCC**GCG**CCCC**GCG**CCCC**GAG**CCCCC**A
iM_4	**CC**T**CC**T**CC**T**CC**T**CC**T**CC**T**CC**T**CC**T-[BtnTg]
G4_1	[Btn]-**GGG**A**GG**CGT**GG**CCT**GGG**C**GGG**ACT**GGGG**
G4_2	[BtnTg]-T**GGGGG**CTC**GGGG**CGC**GGGG**CGC**GGGG**CAT**GGGG**C
Control_1	CAATCTCAATCTCAATCTCAATCT-[BtnTg]
Control_2	[Btn]-GGGGACTTTCCAGGGAGGCGTGGCCTGTGCGTGACTGGGGAGTGG

Table S3. iM peaks, unique and shared with G4 peaks, organized according to SEACR peak signal.

	signal range*	iM-peaks	peaks in iM-unique	peaks in G4/iM-shared
**HEK293T**	bottom	5309	3332	1977
	middle	10621	2379	8248
	top	5310	16	5294
**WDLPS**	bottom	192	107	85
	middle	382	161	221
	top	192	22	170

*bottom ≤ 1st quartile; middle: interquartile range; top ≥ 3rd quartile

Table S4. G4 peaks, unique and shared with iM peaks, organized according to SEACR peak signal.

	signal range*	G4-peaks	peaks in G4-unique	peaks in G4/iM-common
**HEK293T**	bottom	6406	5272	1134
	middle	12801	5625	7176
	top	6404	118	6286
**WDLPS**	bottom	728	700	28
	middle	1456	1311	145
	top	728	433	295

*bottom ≤ 1st quartile; middle: interquartile range; top ≥ 3rd quartile

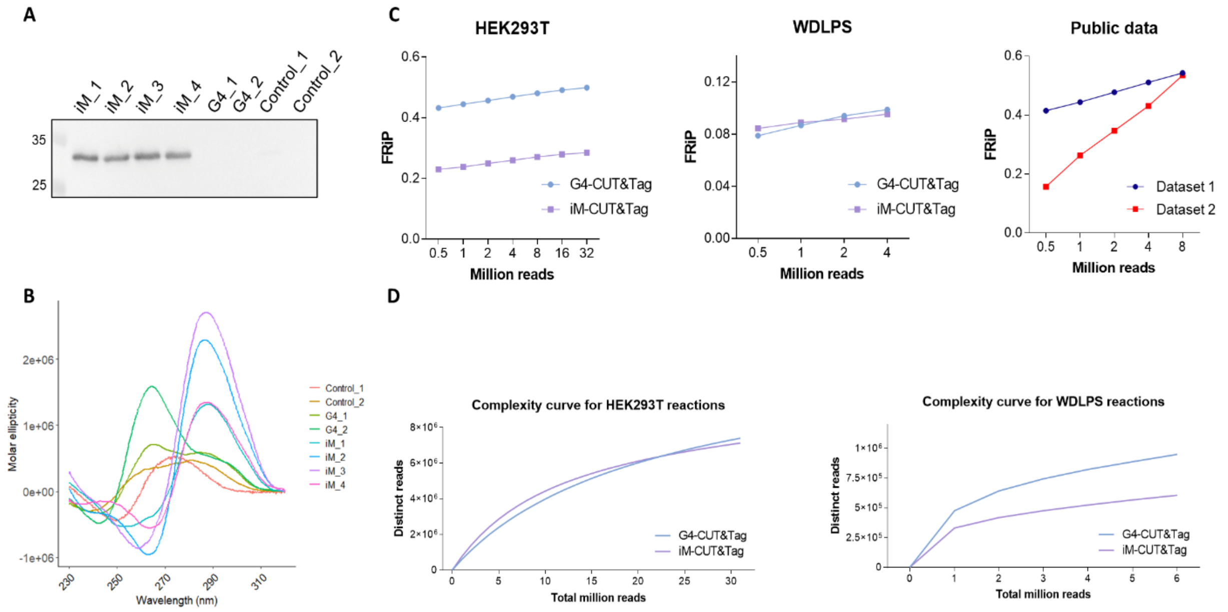

Figure S1. A) Western blot with the anti-FLAG antibody, following iMab pull-down with iMs (iM_1, iM_2, iM_3, iM_4), G4s (G4_1, G4_2) and unstructured (Control_1, Control_2) biotinylated oligonucleotides. B) CD spectra of the biotinylated sequences used for the assay in A). C) Saturation curves for HEK293T, WDLPS and deposited data (dataset 1 (1), dataset 2 (2)) reactions: G4- (light blue) and iM-CUT&Tag (light purple). The mean number of reads derived from the available biological replicates, falling within the called peaks, were plotted. D) G4- (light blue) and iM-CUT&Tag (light purple) complexity curves derived from preseq analysis (3). Mean values among replicates are reported.

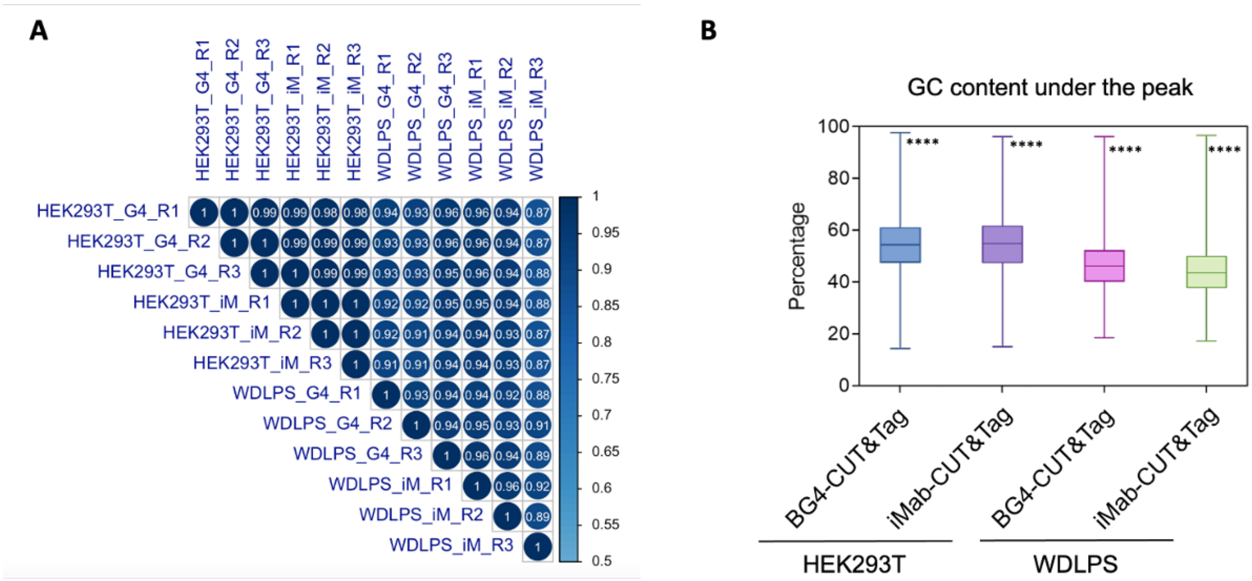

Figure S2. A) Correlation plot for CUT&Tag biological replicates. For all samples, the Pearson's correlation value is reported. B) Percentage of GC bases in iM and G4 peaks. Data are shown for both cell lines: HEK293T (left) and WDLPS (right). A two-sided t-test (CI 95%) was used to assess the statistical significance by comparing the GC content of the CUT&Tag peaks with respect to the GC genomic abundance (average 41%). P-value < 0.0001 (****).

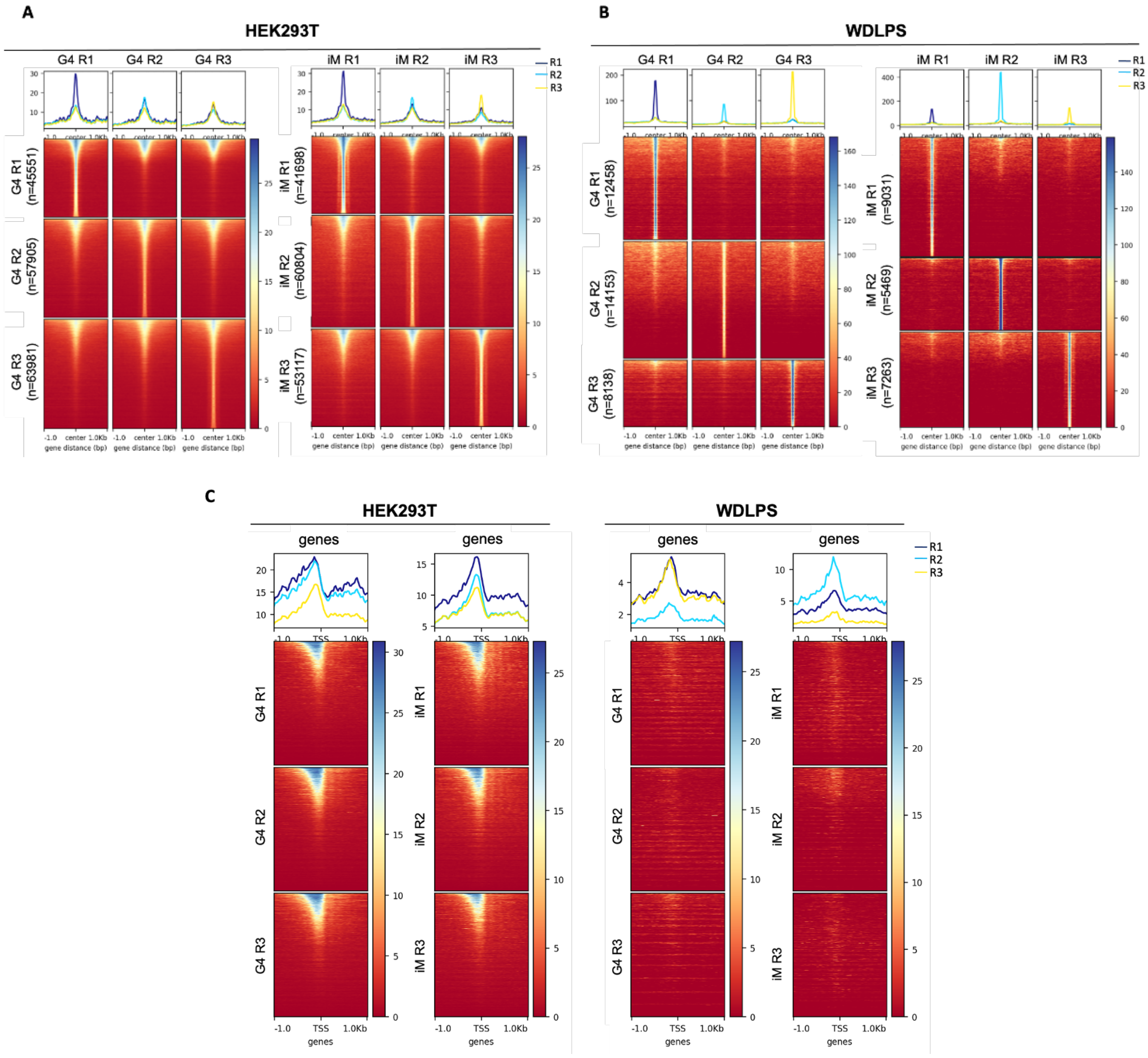

Figure S3. Distribution of the CUT&Tag peaks between replicates (R1, R2, R3). Average plots (top panel) and density heatmaps (bottom panel) of G4 and iM CUT&Tag reads in HEK293T (A) and WDLPS (B) cells. For each cell line, the correlation between biological replicates is referred to the center of the peak, within ± 1 kb distance. (C) Correlation between biological replicates is referred to the transcription start site (TSS), within ± 1 kb distance (n=207289). Y-axes report the mean intensity of the read coverage of the size-normalized library in the displayed regions. Values have a wide range (from 6 to 400 in the different panels) because only the regions corresponding to the called peaks, thus high coverage regions, are reported in panels A and B. In contrast, in panel C, the TSS of all genes are used as a reference point: as they account for genes both with and without peaks, the overall mean intensity is lower.

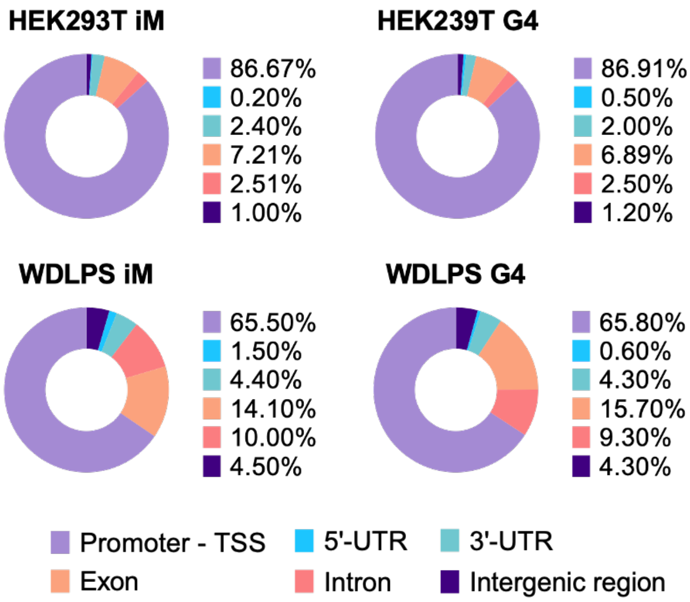

Figure S4. Donut chart showing the distribution of unique peaks among replicates for both G4- and iM-peaks in functional genomic regions according to Chipseeker annotation. Percentages are normalized over the genomic abundance of each functional region.

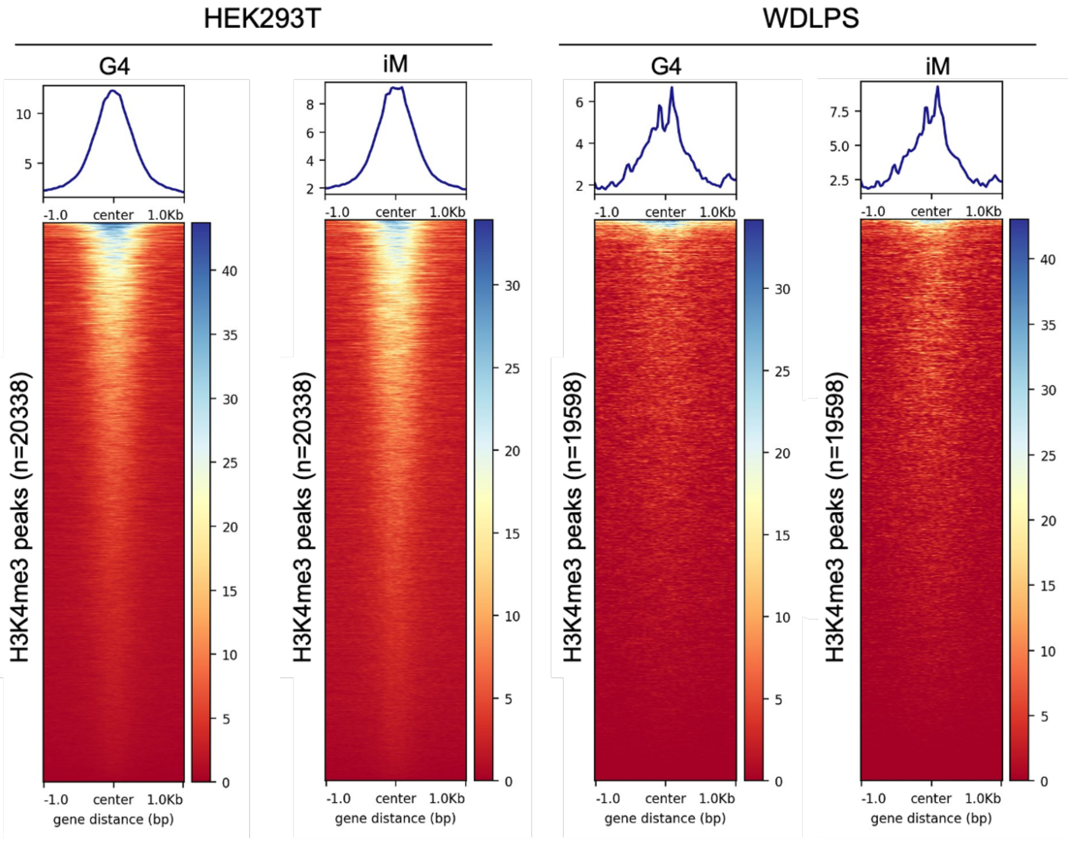

Figure S5. Density heatmaps showing coverage of G4 and iM-CUT&Tag reads in HEK293T (left) and WDLPS (right) cell lines. Signals are referred to active chromatin sites defined by H3K4me3-CUT&Tag reaction.

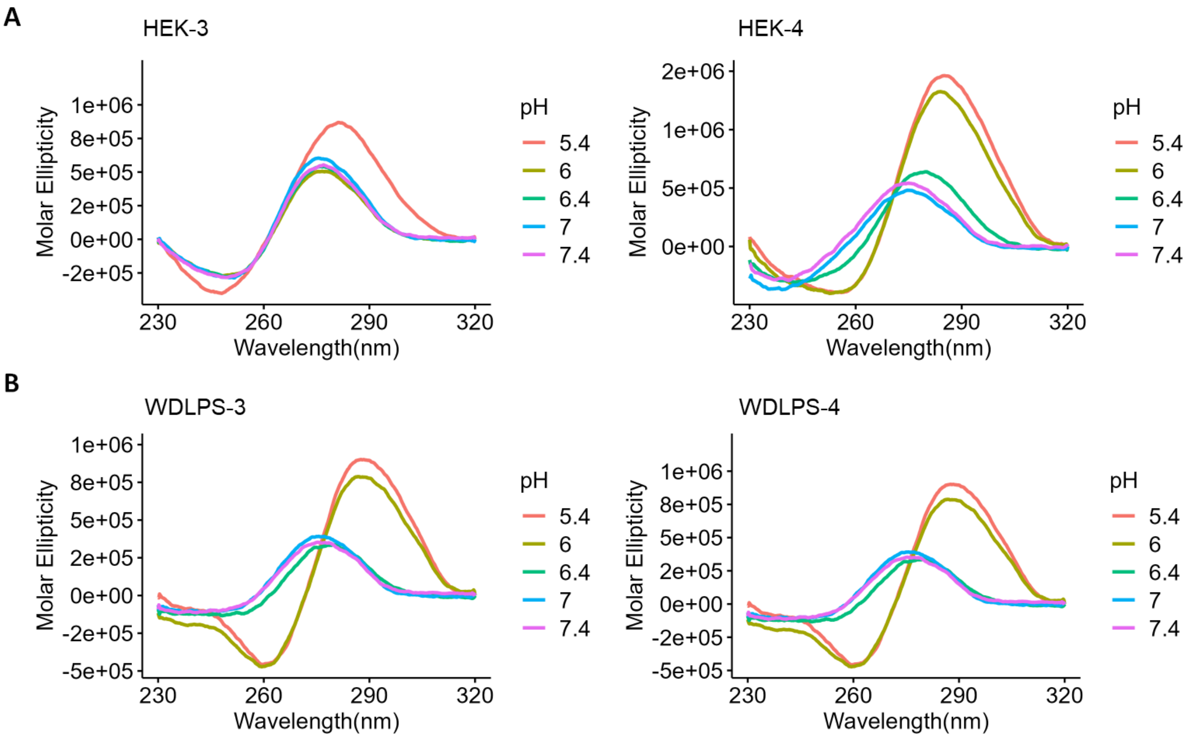

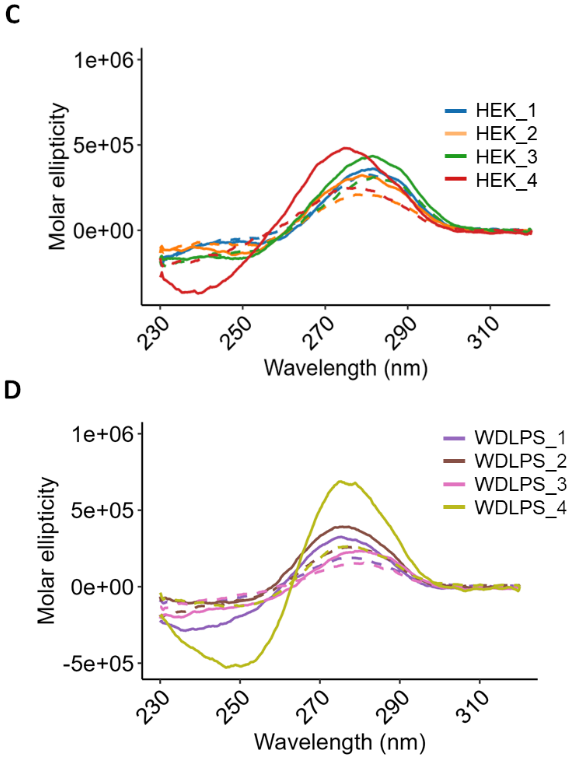

	pH 5.4		pH 7.4	
**Sequence**	**λmin**	**λmax**	**λmin**	**λmax**
**HEK-1**	265.6	289.0	230.0	280.0
**HEK-2**	261.2	287.6	249.4	279.6
**HEK-3**	265.6	288.4	248.4	279.6
**HEK-4**	256.0	285.0	241.0	275.0
**WDLPS-1**	257.2	286.4	240.4	275.6
**WDLPS-2**	259.4	287.6	235.8	275.4
**WDLPS-3**	240.4	289.8	230.6	280.2
**WDLPS-4**	248.0	281.2	249.2	277.0

Figure S6. CD spectroscopy analysis of representative iMs in HEK293T (A) and WDLPS (B) cells. Oligonucleotides were folded in phosphate buffer and analyzed at different pH levels, as indicated. For each sequence, minimum and maximum peaks wavelength at pH 5.4 and 7.4 are reported in the table. CD analysis of HEK (C) and WDLPS (D) iM-forming sequences performed at pH 7 in the presence (plain line) or absence (dashed line) of PEG200 40% (v/v).

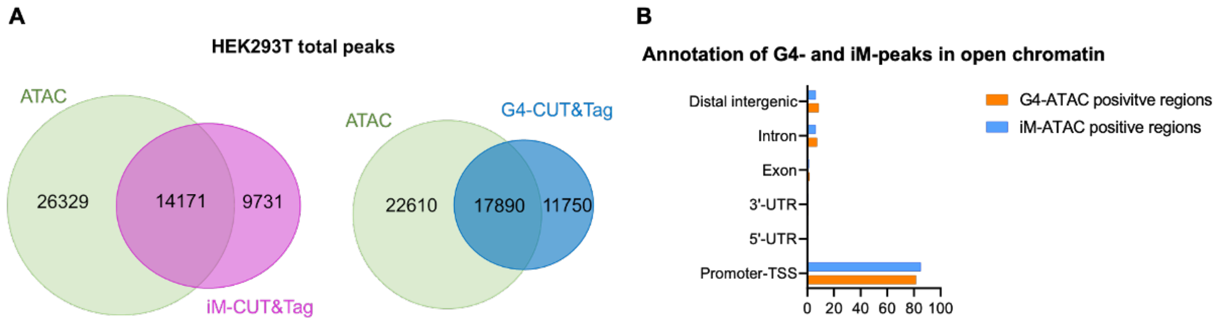

Figure S7. A) Venn diagrams showing shared peaks between ATAC-seq (green) and iM- (purple) or G4-CUT&Tag samples (blu). B) ChIPseeker annotation of G4- (orange) and iM-CUT&Tag (blue) peaks shared with ATAC-seq. Values are reported as percentage distribution in the main genome categories.

Supplementary references

1. Hui,W.W.I., Simeone,A., Zyner,K.G., Tannahill,D. and Balasubramanian,S. (2021) Single-cell mapping of DNA G-quadruplex structures in human cancer cells. *Sci Rep*, 11, 23641.

2. Lyu,J., Shao,R., Kwong Yung,P.Y. and Elsässer,S.J. (2022) Genome-wide mapping of G-quadruplex structures with CUT&Tag. *Nucleic Acids Research*, 50, e13.

3. Daley,T. and Smith,A.D. (2013) Predicting the molecular complexity of sequencing libraries. *Nat Methods*, 10, 325–327.
